# Supplementary material for: Impact of feeding strategies on the welfare and behaviour of horses in groups: An experimental study
Source: PLoS One. 2025 Jun 25;20(6):e0325928. doi: 10.1371/journal.pone.0325928 (PMC12193674; doi:10.1371/journal.pone.0325928)
Supplement: S1 Table — (PDF) [file pone.0325928.s001.pdf]

Impact of feeding strategies on the welfare and behaviour of horses in groups: an experimental study

Marie Roig-Pons, Iris Bachmann, Sabrina Briefer Freymond

Supporting Information

S1 Table

	**Repeats**			
**Group**	*Rep. 0 (no data collection)*	*Rep. 1*	*Rep. 2*	*Rep. 3*
Group 1	*SF*	Portioned	Traditional	SF
Group 2	*SF*	Portioned	SF	Traditional
Group 3	*Portioned*	Traditional	Portioned	SF
Group 4	*Traditional*	SF	Traditional	Portioned

S1 Table – Cross-over design used in a feeding management study, carried out on 18 mares divided into four groups. The Repeat 0 was created to have a balanced plan, but no data was collected. Traditional: Hay available in three 2-hour slots daily (7-9 am, 1-3 pm, 7-9 pm), totalling 6 hours. Portioned: Hay available in six 1-hour slots daily (3-4 am, 7-8 am, 11-12 pm, 3-4 pm, 7-8 pm, 11-12 pm), also totalling 6 hours. *Slow-feeding* with hay nets: Ad libitum hay with one side of the rack open and covered by a 40mm mesh hay net.
