# Supplementary material for: Impact of feeding strategies on the welfare and behaviour of horses in groups: An experimental study
Source: PLoS One. 2025 Jun 25;20(6):e0325928. doi: 10.1371/journal.pone.0325928 (PMC12193674; doi:10.1371/journal.pone.0325928)
Supplement: S2 Table — Each behaviour (with its valence and type) is described in detail, together with its reference if necessary. (PDF) [file pone.0325928.s002.pdf]

Impact of feeding strategies on the welfare and behaviour of horses in groups: an experimental study

Marie Roig-Pons, Iris Bachmann, Sabrina Briefer Freymond

Supporting Information

S2 Table

S2 Table– Detailed ethogram used for the continuous observations. Each behaviour (with its valence and type) is described in detail, together with its reference if necessary.

This ethogram is based on Heitor et al. (2006), Jorgensen et al. (2009) Burla et al. (2016) [1–3]

Valence of the interaction	Type of interactions	Behaviour	Description of the behaviour
Affiliative interactions	Movement	Follow	Horse A moves immediately behind another horse (Horse B) that has just started moving and is less than three body lengths away, for at least 10 seconds without making physical contact with it (Heitor et al., 2006)
	Proximity	Approaches (followed by contact or standing rest nearby)	Horse A moves within two body lengths of another horse (Horse B) that does not immediately move away and remain there for at least 10 seconds with or without establishing physical contact with it (Heitor et al., 2006)
	Approach for social interactions	Contact	Physical contact initiated by Horse A with Horse B.
		Allo-grooming	Mutual grooming in which the partners stand next to each other, usually with their head against the shoulder or the head against the hindquarters, and groom each other's neck, mane, croup, or tail by nibbling or rubbing gently (Jorgensen et al., 2009)
		Play	Play directed towards another individual, who may or may not reciprocate; includes low-intensity play movements such as nipping, grasping, and pulling the mane or tail.
Agonistic interactions	Passive displacements	Passive displacement	The approach of a horse with ears pointed forward or laterally is followed by another horse that moves away. (Burla et al., 2016)
	Push	Push	Pressing the head, neck, shoulder, chest, or body against another horse, forcing it to move one or more limbs to maintain balance. (Jorgensen et al., 2009)

	Threatening behaviours	Back	Moving backwards towards another horse with ears pointing backward. (Jorgensen et al., 2009)
		Head threat	Extending the head and neck towards another horse with ears flattened backward. (Burla et al., 2016)
		Threat to bite	Biting movement, performed by rapidly opening and closing the mouth, with an extension of the neck and ears held back, but without physical contact. (Burla et al., 2016)
		Threat to kick	Kicking movement, executed by rotating the croup or stepping backward, and swinging or striking the hind leg towards another horse, without physical contact. (Burla et al., 2016)
	Aggressive behaviours	Bite	Rapid opening and closing of the jaws when in physical contact with the body of another horse; the ears are flattened backward and the lips retract. (Burla et al., 2016)
		Kick	One or both hind legs are lifted off the ground and rapidly extended backwards towards another horse. (Burla et al., 2016)
		Attack	Rapid movement of Horse A towards Horse B followed by a bite.
		Chase	One horse chases another (at a trot or canter) with ears flattened back and sometimes with the neck extended and the muscles of the muzzle contracted. (Burla et al., 2016)

References

[1] Heitor F, Oom M do M, Vicente L. Social relationships in a herd of Sorraia horses: Part I. Correlates of social dominance and contexts of aggression. Behav Processes 2006;73:170–7. https://doi.org/10.1016/j.beproc.2006.05.004.

[2] Jørgensen GHM, Borsheim L, Mejdell CM, Søndergaard E, Bøe KE. Grouping horses according to gender—effects on aggression, spacing and injuries. Appl Anim Behav Sci 2009;120:94–9.

[3] Burla J-B, Ostertag A, Patt A, Bachmann I, Hillmann E. Effects of feeding management and group composition on agonistic behaviour of group-housed horses. Appl Anim Behav Sci 2016;176:32–42.
